# Population pharmacokinetics and dose optimization of piperacillin-tazobactam in premature and term neonates with severe infections

**DOI:** 10.1128/aac.00998-25

**Published:** 2025-11-25

**Authors:** Frida S. Boer-Pérez, Victoria Lima-Rogel, Silvia Romano-Moreno, Ana R. Mejía-Elizondo, Susanna E. Medellín-Garibay, Paula Schaiquevich, Daniel E. Noyola-Cherpitel, Ana S. Rodríguez-Báez, Cristian J. Rodríguez-Pinal, Rosa del C. Milán-Segovia

**Affiliations:** 1 Facultad de Ciencias Químicas, Universidad Autónoma de San Luis Potosí 439402 , San Luis Potosí, Mexico; 2 Neonatal Intensive Care Unit, Hospital Central “Dr. Ignacio Morones Prieto” 61727, San Luis Potosí, Mexico; 3 Unit of Innovative Treatments, Hospital de Pediatria JP Garrahan, Buenos Aires, Argentina; 4 National Council of Scientific and Technical Research (CONICET), Buenos Aires, Argentina; 5 Microbiology Department, Facultad de Medicina, Universidad Autónoma de San Luis Potosí 84969 , San Luis Potosí, Mexico; 6 Department of Clinical Pharmacy, Institute of Pharmacy, University of Bonn 9374, Bonn, Germany; Providence Portland Medical Center, Portland, Oregon, USA

**Keywords:** neonates, piperacillin, antibiotics, individualized drug therapy, population pharmacokinetics

## Abstract

Piperacillin-tazobactam is widely used off-label in neonates for the empirical treatment of severe infections, resulting in diverse dosing regimens across clinical settings. This variability, combined with high interindividual differences in renal maturation that impact drug disposition, complicates standardized dosing and emphasizes the need for individualized, evidence-based strategies. This study aimed to develop and evaluate a population pharmacokinetic model of piperacillin in neonates to support optimized initial dosing recommendations. Neonatal patients (postnatal age ≤28 days) admitted to an intensive care unit who received piperacillin-tazobactam (8:1 ratio) at Neofax-recommended doses were included. Plasma concentrations were measured using an ultra-high-performance liquid chromatography with tandem mass spectrometry method. Population pharmacokinetic analysis for piperacillin was performed using nonlinear mixed-effects modeling. A total of 65 blood samples were collected from 25 neonates, both preterm (56%) and full-term. Piperacillin pharmacokinetics was best described by a one-compartment model incorporating body weight-based allometric scaling, postmenstrual age, and serum creatinine as covariates influencing clearance. For a typical neonate in the study (1.76 kg), the estimated clearance was 0.748 L/h (coefficient of variation, 38.3%), and the volume of distribution was 0.866 L (37.7%). Simulations indicated adequate probability of target attainment with current recommendations, but a high proportion of preterm neonates (>75%) were at risk of overexposure (trough piperacillin plasma concentration >50 mg/L). Additional simulations supported individualized initial regimens based on renal maturation and suggested that extended infusions (1−4 h) may improve target attainment for stricter targets and less-susceptible pathogens. This study provides a validated pharmacokinetic model for piperacillin in neonates with severe infections, offering a framework to optimize empiric dosing based on renal function and developmental stage.

## INTRODUCTION

Antibiotics are among the most administered therapies in neonatal intensive care units (NICUs), where hospitalized neonates, particularly those born preterm, are highly vulnerable to infectious complications due to their immature immune systems and the frequent need for invasive medical interventions (1, 2). Neonatal sepsis continues to represent a significant global health concern, with an estimated 1.3 to 3.9 million annual cases worldwide, according to the World Health Organization, and remains a significant cause of neonatal mortality (3, 4). Late-onset sepsis (LOS)—defined as sepsis occurring after 72 h of life—is a frequent and clinically important complication in this population (5). LOS is primarily associated with horizontal transmission of pathogens from caregivers or the hospital environment and is particularly prevalent among preterm infants requiring prolonged respiratory support, parenteral nutrition, and central venous access (6–9).

Piperacillin, a broad-spectrum β-lactam antibiotic, is commonly administered in combination with tazobactam, a β-lactamase inhibitor, for the empirical treatment of severe and complicated infections in neonates (10–12). Although piperacillin-tazobactam is approved for pediatric use in infants older than 2 months, its administration in neonates remains off-label and is clinically justified due to the high risk of morbidity and mortality associated with untreated serious infections in this vulnerable population (13–16). In clinical practice, dosing is typically guided by references such as Neofax and The Harriet Lane Handbook, which are based on limited pharmacokinetic data in neonates and young infants (17, 18). Additionally, individual hospitals may implement their own dosing protocols, further contributing to the variability observed in neonatal antibiotic regimens (19–21).

From a pharmacokinetic and pharmacodynamic (PK-PD) perspective, β-lactams demonstrate time-dependent antibacterial activity (22). In this context, the time that free serum concentrations remain above the minimum inhibitory concentration (MIC) during the dosing interval (%fT>MIC) serves as a surrogate marker for the effectiveness of the antimicrobial treatment. Although recent studies have also explored PK-PD targets for tazobactam, dosing of this combination is based on the fixed ratio of both agents, and tazobactam cannot be administered independently. In pediatric patients, particularly preterm neonates, PK-PD thresholds remain heterogeneous and are extrapolated from adults without validation in neonatal patients (23, 24). The US Food and Drug Administration generally recommends a target of ≥50% fT>MIC (25, 26). In contrast, other studies suggest that preterm neonates in the NICU may require stricter thresholds, such as ≥75% fT>MIC, due to their underdeveloped immune systems (27, 28).

Optimizing antibiotic therapy in NICU patients is challenged by substantial pharmacokinetic variability driven by rapid developmental changes in organ function and the impact of critical illness (29–31). In this context, pharmacokinetic approaches are crucial for guiding evidence-based dosing strategies that achieve a balance between efficacy and safety (32–34). Population pharmacokinetic modeling allows the incorporation of developmental covariates, such as gestational and postmenstrual age (GA and PMA), to capture maturational trends in elimination pathways (24). These models, when combined with Monte Carlo simulations, support the identification of optimized dosing regimens for defined neonatal subgroups, enabling more precise and individualized antibiotic therapy.

The purpose of this study was to characterize the population pharmacokinetics of piperacillin in preterm and full-term neonates with LOS or other severe infections and to evaluate the influence of clinical, physiological, and maturational factors on drug disposition. The second objective was to assess the probability of target attainment (PTA) for two PK-PD thresholds (≥50% fT>MIC and ≥75% fT>MIC) and to evaluate the risk of overexposure using a free trough concentration of 50 mg/L as an exploratory safety threshold to propose optimized dosing strategies for this population.

## RESULTS

### Study population

A total of 25 neonatal patients were included in the model development process, and eight additional neonates comprised the external validation cohort used to assess the predictive performance of the final population pharmacokinetic model. Demographic, anthropometric, and clinical characteristics, including co-administered medications, are summarized in Table 1. In the model development group, 56% of neonates were preterm, a proportion comparable to that in the validation group (50%).

**TABLE 1:** Demographic and clinical details of neonates enrolled in the study^*a*^

Characteristics	Model development		External validation	
	Mean (SD)	Median (range)	Mean (SD)	Median (range)
**Demographic details**				
Female (*n* [%])		14 (56%)		3 (37%)
GA (weeks)	34.3 (4.6)	34.2 (26−41.1)	35.3 (3.8)	35.3 (30.5−40.9)
PNA (days)	15.7 (6.4)	14 (6−28)	13.9 (6)	12 (8−27)
PMA (weeks)	36.5 (4.3)	36.0 (28.1−43.3)	37.1 (4.0)	37.2 (32.2−42.8)
BW (kg)	1.97 (0.80)	1.76 (0.89−3.57)	1.99 (0.86)	1.64 (1.14−3.46)
Height (cm)	42 (5)	42 (31−55)	44 (5)	44 (35−52)
BSA (m^2^)	0.15 (0.04)	0.14 (0.09−0.22)	0.16 (0.04)	0.14 (0.11−0.22)
**Laboratory values**				
SCr (mg/dL)	0.45 (0.15)	0.4 (0.2−0.9)	0.47 (0.12)	0.4 (0.3−0.6)
CL_Cr_ (mL/min/1.73 m^2^)	40.4 (22.2)	32 (15−103)	36.6 (17.4)	35 (19−76)
Total bilirubin (mg/dL)	6.2 (5.8)	4.4 (0.1−18.4)	5.1 (4.3)	4.5 (1.1−11.8)
Total proteins (g/dL)	4.7 (0.7)	4.9 (3.3−6.3)	4.7 (0.4)	4.5 (4.3−5.3)
Albumin (g/dL)	2.6 (0.5)	2.5 (1.7−3.7)	2.9 (0.5)	2.7 (2.3−3.5)
AST (U/L)	48 (49)	34 (15−241)	31 (20)	22 (16−62)
ALT (U/L)	24 (34)	11 (5−136)	15 (10)	9 (4–31)
N**eonatal category,** ***n*** **(%)**				
Extremely preterm		1 (4%)		0 (0%)
Moderate–late preterm		13 (52%)		4 (50%)
Term		11 (44%)		4 (50%)
**Clinical data and co-administration drugs,** ***n*** **(%)**				
Mechanical ventilation		7 (2.8%)		4 (50%)
Sepsis		12 (48%)		5 (62%)
Vancomycin		2 (8%)		1 (12%)
Levetiracetam		6 (24%)		1 (12%)
Caffeine		10 (40%)		1 (12%)
Paracetamol		4 (16%)		0 (0%)

^*a*^ SD: standard deviation; GA: gestational age; PNA: postnatal age; PMA: postmenstrual age; BW: body weight; BSA, body surface area; SCr: serum creatinine; CL_Cr_: creatinine clearance; AST: aspartate aminotransferase; ALT: alanine aminotransferase.

LOS was the most common indication for piperacillin-tazobactam therapy in this cohort, followed by necrotizing enterocolitis (16%, Bell stage II or higher) and healthcare-associated pneumonia (16%). For model development, a total of 65 piperacillin plasma concentrations were included, with a median (range) of three (2–3) samples per patient. Most samples (60%) were obtained during the first 2 weeks of life, and all were collected after administration of at least three doses of piperacillin-tazobactam. Piperacillin plasma concentrations ranged from 5.7 to 302.6 mg/L, while tazobactam concentrations ranged from 1.1 to 38.0 mg/L. One tazobactam concentration (0.4 mg/L) was below the lower limit of quantification (LLOQ = 0.6 mg/L) and was therefore excluded from all analyses. The distribution of plasma concentrations for both drugs is shown in Fig. S1. The mean piperacillin-tazobactam plasma concentration ratio was 11.8:1 (±4.2:1), reflecting the relationship between the two components in neonatal patients. Ratios varied across patients and appeared to increase over time in those with higher PMA, although in some patients, the ratio remained relatively stable throughout the dosing interval (Fig. S2).

### Pharmacokinetic analysis

A one-compartment model with first-order elimination adequately described the piperacillin data. Interindividual variability (IIV) was estimated for both clearance (CL) and volume of distribution (V) using an exponential (log-normal) model, while a proportional error model best characterized residual unexplained variability.

Body weight (BW), PMA, and serum creatinine (SCr) were identified as covariates influencing CL, while V was associated only with BW. The effect of PMA on CL was incorporated through a sigmoidal maturation function, with parameters reported by Rhodin et al. (35).

No categorical covariates had a significant influence on CL or V. The final model explained 46.7% of the IIV in CL and 15.3% of the IIV in V. The final covariate equations, population pharmacokinetic parameter estimates, and their precision are summarized in Table 2.

**TABLE 2:** Pharmacokinetic parameters, interindividual variability, and residual error obtained for the piperacillin final model in neonates with severe infections^*a*^

Parameter	Estimate	(RSE %)	(Shrinkage %)	Bootstrap estimates (*n* = 1,000)		
				Median	2.5%	97.5%
**Structural model parameters**						
$CL=\theta_{CL}\times \;{\left(\frac{BW}{1.76}\right)}^{0.75}\times \;\left(\frac{PMA^{Hill}}{PMA^{Hill}+TM_{50}^{Hill}}\right)\times \;{\left(\frac{SCr}{0.4}\right)}^{\theta_{SCr}}$						
$\theta_{CL}$ (L/h)	0.748	(8%)		0.744	0.652	0.850
$\theta_{SCR}$	−0.635	(36%)		−0.604	−0.951	−0.132
$V=\theta_{V}\times {\left(\frac{BW}{1.76}\right)}^{1}$						
$\theta_{V}\;(L)$	0.866	(8%)		0.865	0.756	0.975
**Interindividual variability (%CV)**						
$\omega_{\text{C}L}$	38.3	(12%)	(2%)	35.3	27.0	42.6
$\omega_{V}$	37.7	(17%)	(6%)	35.2	24.2	44.8
**Residual variability (%CV)**						
$\sigma_{proportional}$	11.4	(36%)	(42%)	11.4	7.7	14.5

^*a*^ RSE: relative standard error; CL: clearance; BW: body weight; PMA: postmenstrual age; Hill: Hill coefficient; TM_50_: maturation half-life; SCr: serum creatinine; V: volume of distribution; $\omega_{CL}$: interindividual variability of clearance; $\omega_{V}$: interindividual variability of volume of distribution; $\sigma_{proportional}$: proportional residual error.

### Model evaluation

The final population pharmacokinetic model for piperacillin demonstrated stability and robustness. All parameter estimates, including IIV terms, fell within the 95% confidence intervals derived from the bootstrap analysis and deviated by less than 9% from the corresponding bootstrap medians, indicating symmetrical distributions and consistent model performance.

**Fig 1:**
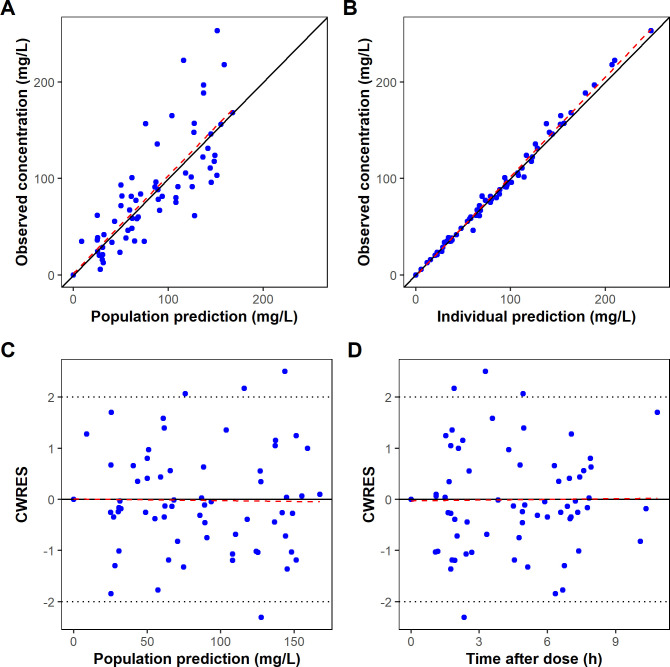
Goodness-of-fit plots for the final piperacillin population pharmacokinetic model. Observed piperacillin concentrations versus (**A**) population predictions and (**B**) individual predictions. Conditional weighted residuals versus (**C**) population predictions and (**D**) time after dose.

**Fig 2:**
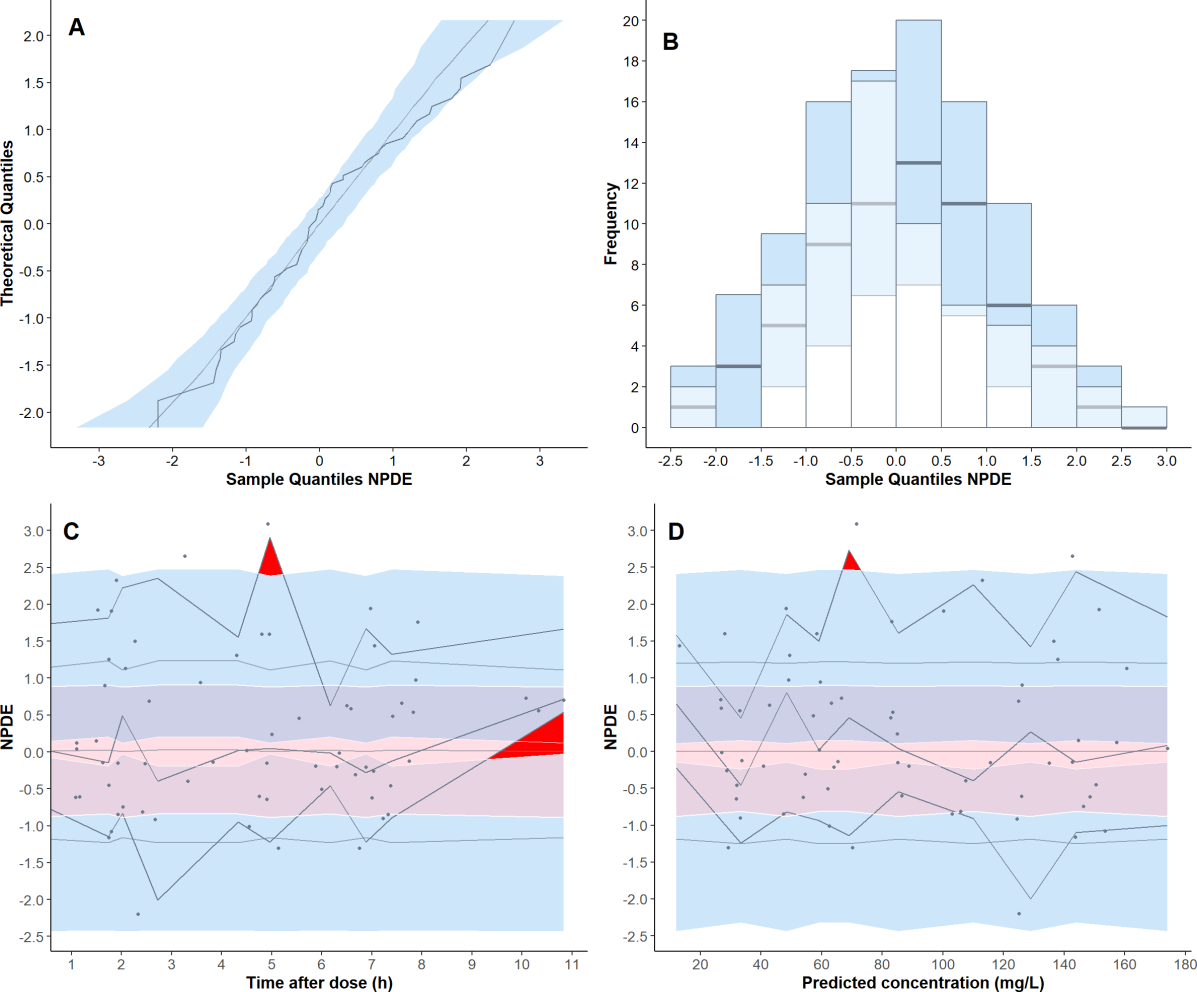
NPDE of the final piperacillin population pharmacokinetic model. (**A**) Quantile-quantile plot of NPDE versus the expected standard normal distribution; (**B**) histogram of NPDE with the density of the standard normal distribution overlaid; (**C**) scatter plot of NPDE versus time after the last dose; (**D**) scatter plot of NPDE versus population prediction. Panels** C **and **D** show the observed piperacillin data as gray circles, with the black solid lines representing the 2.5th percentile, median, and 97.5th percentile of the observed data. Shaded areas indicate the 95% confidence intervals of the median (red) and percentiles (blue).

**Fig 3:**
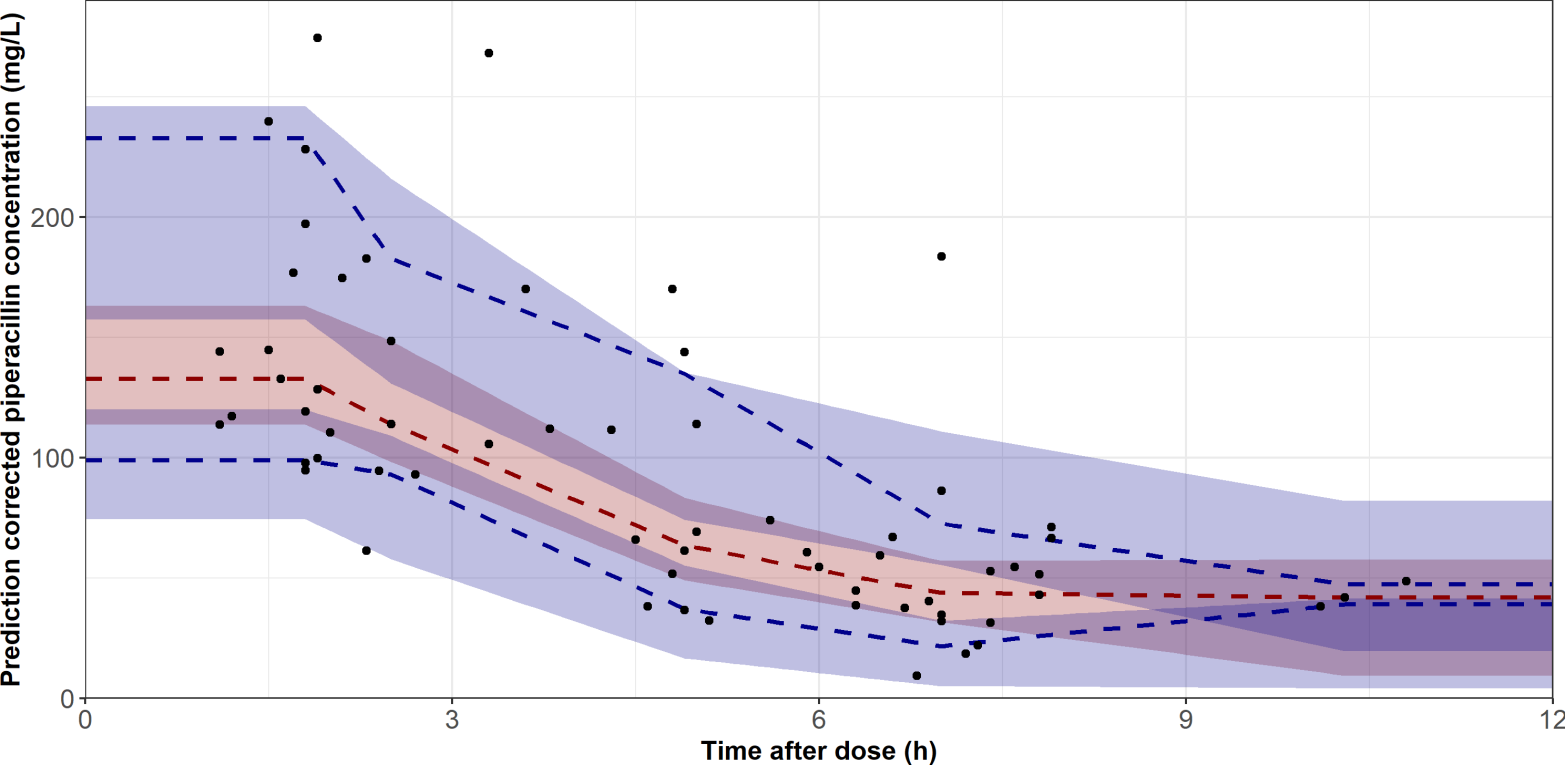
VPC for piperacillin plasma concentrations based on 1,000 simulations. Dashed lines represent the observed median (red) and the 5th and 95th percentiles (blue). Shaded areas indicate the 90% confidence intervals of the simulated median (red) and percentiles (blue). The circles represent the observed piperacillin data.

Goodness-of-fit diagnostics showed satisfactory agreement between observed and both population and individual predicted plasma concentrations (Fig. 1A and B). Conditional weighted residuals were symmetrically distributed around zero, with no evident trends across predicted concentrations or time since last dose (Fig. 1C and D). The normalized prediction distribution error (NPDE) analysis results are presented in Fig. 2. The NPDE values exhibited a normal distribution and density (Fig. 2A and B). They were well distributed over time and across predicted piperacillin concentrations (Fig. 2C and D). Although two values fell outside the upper percentile confidence interval, no significant trends were observed. The global normality test for the final piperacillin model corresponded to a *P*-value of 0.409, indicating that the model predictions were consistent with the expected variability. Consistently, the prediction-corrected visual predictive check (pcVPC) results confirmed the model’s predictive accuracy in the neonatal population (Fig. 3).

The external evaluation demonstrated that the final model accurately characterized the pharmacokinetic behavior of piperacillin in neonates (Table 3). The mean prediction error (MPE) was 2.29 mg/L with a 95% confidence interval of −6.27 to 12.5 mg/L, while the mean absolute prediction error (MAPE) was 25.89%, indicating low bias and acceptable accuracy. The root mean squared error (RMSE) was 17.75 mg/L, corresponding to approximately 23% of the mean observed concentrations in the external data set, supporting the model’s overall predictive precision in this population. Moreover, the external pcVPC showed that the observed concentrations fell within the 95% confidence intervals, with the median trend of the observations closely following the model-predicted median (Fig. S3). No systematic trends were evident, further supporting the model’s ability to describe the external neonatal data set.

**TABLE 3:** External validation analysis for the piperacillin final model^*a*^

Parameter	Absolute value (mg/L)	95% CI (mg/L)	Relative value (%)
MPE	2.29	−6.27 to 12.5	13.23
MAPE	14.57		25.89
Root mean squared error (RMSE)	17.75		22.92

^*a*^ 95% CI: 95% confidence interval.

### Monte Carlo simulations for evaluation and optimization of dosing regimens

The performance of existing piperacillin-tazobactam dosing regimens recommended by Neofax and Harriet Lane Handbook was evaluated in virtual neonatal populations grouped by PMA, based on the PTA and the risk of drug overexposure. At an MIC of 16 mg/L, corresponding to *Enterobacterales*, both regimens achieved >90% PTA for PK-PD targets of 50% and 75% fT>MIC across all subgroups. At an MIC of 32 mg/L for *Pseudomonas aeruginosa*, PTA remained above 90% when the 50% fT>MIC threshold was applied for both regimens. However, under the stricter 75% fT>MIC target, the Neofax regimen demonstrated a substantial decline in PTA, dropping to 68% in moderate-to-late preterm neonates and 37% in term neonates with SCr <0.4 mg/dL. In contrast, the Harriet Lane Handbook regimen consistently achieved >80% PTA across all gestational and renal function subgroups at both PK-PD targets, including at the higher MIC of 32 mg/L.

Despite meeting efficacy targets, both existing regimens were associated with high rates of potentially toxic trough concentrations. Among preterm neonates, more than 75% exceeded the safety threshold, with rates reaching 100% in the extremely preterm group. In term neonates, overexposure rates ranged from 33.7% to 65.8%, depending on renal function, with lower frequencies observed in those with lower SCr. In contrast, the optimized dosing regimens proposed in this study achieved >90% PTA at 16 mg/L and >80% at 32 mg/L for both PK-PD targets, while reducing the proportion of patients exceeding the trough safety threshold to approximately 30% across all subgroups (Table 4).

**TABLE 4:** Evaluation of piperacillin-tazobactam regimens in neonates: simulated probability of target attainment and toxicity risk across PMA and SCr subgroups^*a*^

PMA (weeks)	SCr (mg/dL)	Regimen	% PTA MIC = 16 mg/L		% Trough > 50 mg/L	% PTA MIC = 32 mg/L		% Trough > 50 mg/L
			50% fT	75% fT		50% fT	75% fT	
≤29	<0.4	Neofax	99.8	94.9	37.3	96.4	79.7	37.3
		Harriet Lane	100.0	99.8	76.5	100.0	97.3	76.5
		Optimized	99.8	94.9	37.3	97.5	80.6	39.1
	0.4–0.8	Neofax	100.0	99.4	77.9	99.6	95.8	77.9
		Harriet Lane	100.0	100.0	94.9	100.0	99.6	94.9
		Optimized	99.6	95.8	36.1	95.0	83.1	37.8
	>0.8–1.2	Neofax	100.0	100.0	93.3	100.0	99.8	93.3
		Harriet Lane	100.0	100.0	99.8	100.0	100.0	99.8
		Optimized	100.0	97	31.6	93.0	80.7	32.5
30–36	<0.4	Neofax	98.7	90	22.4	93	68	22.4
		Harriet Lane	99.6	95.9	36.8	97.1	82.3	36.8
		Optimized	95.5	93.3	30.4	96.6	79.3	33.1
	0.4–0.8	Neofax	99.9	98.5	62.2	99.1	92.4	62.2
		Harriet Lane	100.0	99.7	77.4	99.7	97	77.4
		Optimized	94.6	90.9	31.6	95.9	77.5	32.2
	>0.8–1.2	Neofax	100.0	100.0	86.9	100.0	99.3	86.9
		Harriet Lane	100.0	100.0	93.3	100.0	100.0	93.3
		Optimized	94.4	93.9	33.6	95.8	80.1	35.1
37–44	<0.4	Neofax	93.1	68.4	6.9	71.0	37	6.9
		Harriet Lane	100.0	98	47.4	98.7	86.8	47.4
		Optimized	100.0	92	22.94	100.0	82.7	29.2
	0.4–0.8	Neofax	99.1	93	33.7	94.8	77.7	33.7
		Harriet Lane	100.0	99.7	86	99.9	98.4	86
		Optimized	99.1	93	33.7	96.2	80.3	35.8
	>0.8–1.2	Neofax	99.9	98.9	65.8	99.5	94.7	65.8
		Harriet Lane	100.0	100.0	97.6	99.9	99.9	97.6
		Optimized	99.1	92.7	28.2	96.4	76.6	29.8

^*a*^ PMA: postmenstrual age; SCr: serum creatinine; PTA: probability of target attainment; Trough: trough plasma concentration. MIC: minimum inhibitory concentration; Neofax: dosing recommendation based on 100 mg/kg every 8 or 12 h, adjusted according to postnatal and postmenstrual age; Harriet Lane: dosing recommendation ranging from 80 to 100 mg/kg every 4, 6, or 8 h, with adjustments based on body weight, postnatal age, and postmenstrual age; Optimized: dosing regimen proposed in this study, ranging from 30 to 130 mg/kg every 8 or 12 h, adjusted for body weight, postmenstrual age, and serum creatinine levels.

These optimized regimens were developed using model-based simulations stratified by PMA and SCr categories to reflect the variability in renal maturation and function among neonates. Intravenous doses ranging from 30 to 130 mg/kg every 8 or 12 h were selected according to PMA and SCr subgroup (Table 5). While dosing was similar for both MIC scenarios (16 and 32 mg/L), longer infusion durations were considered necessary to enhance target attainment for the treatment of infections caused by organisms with a higher MIC. By adjusting both dose and dosing interval based on developmental stage and renal function, the optimized regimens demonstrated favorable target attainment profiles and reduced overexposure risk in simulated neonates, suggesting potential clinical benefits in this population.

**TABLE 5:** Dosing recommendations of piperacillin-tazobactam for neonates with severe infections based on simulations of the final pharmacokinetic model to achieve a PTA >90% for an MIC = 16 mg/L and >80% for an MIC = 32 mg/L^*a*^

PMA (weeks)	SCr (mg/dL)	Dose	MIC = 16 mg/L	MIC = 32 mg/L
			Infusion duration (h)	Infusion duration (h)
≤29	<0.4	100 mg/kg q12 h	0.5	1.0
	0.4−0.8	50 mg/kg q12 h	0.5	1.0
	>0.8−1.2	30 mg/kg q12 h	0.5	1.0
30−36	<0.4	120 mg/kg q8 h	0.5	1.0
	0.4−0.8	120 mg/kg q12 h	0.5	1.0
	>0.8−1.2	70 mg/kg q12 h	0.5	1.0
37−44	<0.4	130 mg/kg q8 h	3.0	4.0
	0.4−0.8	100 mg/kg q8 h	0.5	1.0
	>0.8−1.2	110 mg/kg q12 h	0.5	1.0

^*a*^ PMA: postmenstrual age; SCr: serum creatinine; MIC: minimum inhibitory concentration.

## DISCUSSION

Piperacillin-tazobactam is widely used in neonatal intensive care units for the empirical treatment of suspected or confirmed severe infections. However, its use in neonates remains off-label, and safety data in this population are still lacking (36–38). In this study, a population pharmacokinetic model for piperacillin in neonates was developed that integrates both maturational factors (BW and PMA) and a renal function covariate (SCr) to explain IIV in drug CL. The findings obtained in this study contribute to a better understanding of piperacillin disposition during the neonatal period and provide a preliminary framework to support dosing optimization in this vulnerable population, balancing both efficacy and safety considerations.

Previous population pharmacokinetic studies have described piperacillin pharmacokinetics in young infants, including neonates, using one- (27, 28) and two-compartment (39, 40) models. In contrast, our analysis focused exclusively on neonates (postnatal age [PNA] ≤28 days), and a one-compartment model provided the best description of the data. The simple model structure may be related to the small number of samples per patient in this study.

The typical piperacillin CL was estimated at 0.748 L/h for a neonate with a median weight of 1.76 kg, which is similar to the 0.732 L/h extrapolated from the allometrically scaled CL reported by Cohen-Wolkowiez et al. (27), who estimated 0.479 L/h/kg^0.75^. In agreement with prior studies, PMA, BW, and SCr were significant covariates on piperacillin CL (27, 28, 39). Incorporating a sigmoidal maturation function based on PMA improved the model’s predictive performance by accounting for the ontogeny of renal elimination pathways. Since piperacillin is primarily eliminated via glomerular filtration and active tubular secretion, and these renal processes continue to mature throughout the first weeks of life, with distinct developmental patterns in preterm versus term neonates, this approach reflects the underlying developmental physiology (41, 42).

To describe the ontogeny of renal CL, we incorporated maturation parameters derived from Rhodin et al. (35), who developed a sigmoidal glomerular filtration maturation model based on neonatal data. This mechanistic approach was previously used in the development of other antibiotic population pharmacokinetic models in neonates (43, 44). Our results align with recent findings by Kong et al. (40), who identified PMA as a significant covariate in a pooled population pharmacokinetic analysis spanning neonates to elderly adults. Their estimated TM_50_ (54.2 weeks) and Hill coefficient (3.35) are comparable to those used in our model (TM_50_ = 47.7 weeks, Hill = 3.4).

For the piperacillin V, BW was the only covariate retained, consistent with previously published pharmacokinetic models. The typical V was estimated to be 0.866 L (0.492 L/kg), which is very close to the 0.42 L/kg reported by Cohen-Wolkowiez et al. in 2014 but differs from the 2.91 L/kg reported by the same group in 2012 (27, 28). These differences may reflect variations in GA and body composition, particularly the higher total body water content in preterm neonates, which increases the apparent V of hydrophilic drugs like piperacillin (30, 45). Additional covariates related to fluid status or protein binding, such as bilirubin, albumin, total protein, mechanical ventilation, and sepsis, were evaluated but did not significantly improve the model.

Covariates related to patients’ co-medications were assessed but showed no significant association with piperacillin V and CL. Although several concomitant drugs were considered, the number of exposed patients was too limited to allow formal covariate analysis. Further studies with larger cohorts are needed to assess these potential interactions with concomitant medications.

In this study, pharmacokinetic modeling focused exclusively on piperacillin, as dose adjustment is only feasible for this component, and PK-PD targets are defined based on its exposure. For descriptive purposes, plasma concentrations of tazobactam were quantified to verify that its exposure remained within an expected range relative to piperacillin. The plasma concentration–time profiles of tazobactam closely mirrored those of piperacillin, which aligns with the similar pharmacokinetic characteristics previously reported for both compounds (46, 47).

The observed mean plasma concentration ratio of piperacillin-tazobactam in our study population showed higher values and greater variability than previously reported by Reed et al. (48). In their single-dose study in children aged 2 months to 12 years, peak ratios of 9.5:1 (±1.7:1) and trough ratios of 7.3:1 (±1.9:1) were observed for a dose of 100/12.5 mg/kg of piperacillin and tazobactam, which corresponds to the same dosing regimen used in our study, with lower ratios at smaller doses.

Several factors may account for these differences. First, our patients were at steady state, which is expected to yield higher ratios compared with single-dose administration, and our average ratio reflects plasma levels from 0.5 to nearly 12 h post-infusion. Second, our cohort comprised neonates, in whom maturation of renal CL affects both drugs differently than in older children. Tazobactam CL may exceed that of piperacillin in this age group, as reported in population studies including infants <2 months (28, 39).

The increase in the ratio observed in some patients with higher PMA may be due to enhanced renal function, resulting in greater tazobactam CL. Although our study is descriptive and these observations should be interpreted cautiously, prolonged infusions might help maintain a more consistent piperacillin-tazobactam ratio in patients with higher PMA.

Prior studies have shown that plasma ratios of combination β-lactams and β-lactam adjuncts in neonates can differ from those observed in older children or adults, primarily due to developmental differences in pharmacokinetics (49, 50). Overall, our findings suggest that extrapolating fixed-dose combinations derived from adults to neonates may present challenges, underscoring the importance of further dedicated evaluations to determine whether current formulations are optimal for this population.

As mentioned before, defining PK-PD targets in neonates remains challenging, as most recommendations are extrapolated from adult or older pediatric populations and have not been specifically validated in neonatal patients (23, 24, 51). In this context, we evaluated both 50% fT>MIC and 75% fT>MIC as potential efficacy thresholds and selected the stricter one to optimize dosing in our neonatal population. This decision was based on the clinical characteristics of preterm neonates, whose immature immune system and the severity of infections make maximizing bacterial coverage particularly important. While higher thresholds, such as 100% fT>MIC or 100% fT>4 ×MIC, are often recommended in critically ill adults and pediatrics, these are not typically applied in neonatal populations (52, 53).

Although the direct link between PK-PD targets and clinical outcomes in neonates remains uncertain, adopting 75% fT>MIC may offer a logical framework to guide dosing optimization in the absence of neonatal-specific efficacy data. At the same time, the potential risk of overexposure underscores the importance of integrating exposure-safety considerations when tailoring antimicrobial therapy in this population.

To our knowledge, this is the first study to systematically evaluate the exposure threshold potentially associated with nephrotoxicity in neonates treated with piperacillin and to leverage this information to inform dose optimization. The exploratory toxicity threshold applied was previously identified in critically ill pediatric populations but has yet to be validated in neonates (54). Nonetheless, its inclusion offers preliminary insights into the delicate balance between achieving therapeutic efficacy and minimizing toxicity in neonatal dosing strategies.

Monte Carlo simulations using a stringent PK-PD target of 75% fT>MIC found that current neonatal dosing recommendations achieve adequate PTA (≥90%) for MICs up to 16 mg/L. However, at MIC = 32 mg/L, PTA dropped below optimal levels in term neonates with low SCr levels (SCr <0.4 mg/dL), and the Neofax regimen outperformed the Harriet Lane Handbook in this subgroup. Notably, despite meeting PK-PD targets, over 75% of preterm neonates (reaching 100% in the extremely preterm group) exceeded the exploratory toxicity threshold, highlighting a potential risk of nephrotoxicity and the importance of individualized approaches for this population.

Model-based optimized dosing regimens were developed and stratified by PMA and SCr to reflect maturational and functional renal variability. These regimens achieved >90% PTA at an MIC of 16 mg/L and >80% at 32 mg/L for both 50% and 75% fT>MIC targets while limiting the proportion of simulated patients exceeding the exploratory trough safety threshold to approximately 30% in all subgroups. The improvements were most notable in preterm neonates, particularly those in the extremely preterm group and those with reduced renal CL, as indicated by elevated SCr levels. The optimization strategy involved adjusting both the dose and dosing intervals (every 8 or 12 h), with infusion durations modified as needed to enhance target attainment at higher MICs.

For an MIC = 32 mg/L, extended infusions significantly improved target attainment across most subgroups, particularly in term neonates with low SCr levels (<0.4 mg/dL). Prolonged infusion has been previously proposed as an effective strategy to optimize β-lactam therapy by enhancing pharmacodynamic target attainment in pediatric and neonatal populations (55, 56). However, its implementation in neonates may be limited by practical challenges such as restricted intravenous access and the risk of drug incompatibilities. Despite these constraints, the potential benefit of improved PK-PD target achievement warrants careful consideration in clinical decision-making.

This study has some limitations. First, for PK-PD target attainment analyses and dosing regimen optimization, unbound piperacillin plasma concentrations were mathematically estimated from total plasma concentrations using the reported unbound fraction, rather than directly measuring free concentrations in plasma or at the site of infection. Albumin levels were not routinely available for all neonates on the day of sampling, so this approach does not capture the substantial variability in albumin concentrations that can occur in neonates. While this indirect approach introduces uncertainty, it has been previously applied in pharmacokinetic studies and is generally considered acceptable for β-lactam antibiotics with low protein binding, such as piperacillin.

Second, the number of neonates in certain subgroups—specifically, extremely preterm neonates (*n* = 1) and those with SCr >0.8–1.2 mg/dL (*n* = 1)—was very limited. Therefore, dosing recommendations for these groups rely primarily on model-based simulations. Finally, MIC values were not prospectively determined from culture-confirmed infections. Instead, simulations used conservative breakpoint values from CLSI guidelines, reflecting a worst-case scenario. This approach mirrors routine clinical practice in neonatal care, where empirical treatment is often required due to challenges in obtaining samples for microbiological cultures, such as the limited blood volume available in neonates (57).

Although the model demonstrated robust predictive performance, including external validation in a limited neonatal cohort, prospective clinical studies are necessary to confirm the efficacy and safety of the proposed dosing regimens. Additionally, as pharmacokinetic variability exists across neonatal populations, clinical centers should validate the model locally to ensure its applicability before implementing it routinely.

## MATERIALS AND METHODS

### Study population

Neonates admitted to the intensive care unit and treated with piperacillin-tazobactam between September 2020 and May 2024 were eligible for inclusion. The inclusion criteria were (i) PNA ≤29 days, (ii) BW ≥850 g on the day of sampling, to ensure that blood sampling volumes remained within safe limits (58–60), and (iii) hematocrit ≥30%.

Clinical data were collected from medical records and included demographic and anthropometric information (GA, PNA, BW, sex, and height [Ht]), laboratory values (SCr, total bilirubin, albumin, total proteins, aspartate aminotransferase [AST], alanine aminotransferase [ALT], complete blood count, serum electrolytes, and microbiological isolates) if available as part of routine medical care, clinical information (patient diagnoses, including sepsis and presence of mechanical ventilation), and concomitant medications. SCr levels in all patients were quantified using an enzymatic assay on an automated analyzer. Variables such as PNA, GA, BW, Ht, and SCr were used to calculate derived parameters, including PMA (defined as the sum of PNA and GA), body surface area (BSA), and creatinine clearance (CL_Cr_). Body surface area was estimated using the Mosteller formula (Equation 1), and CL_Cr_ was calculated using the Schwartz formula (Equation 2), where *k* takes a value of 0.45 for term neonates and 0.33 for preterm neonates (61, 62).


(1) $$BSA\;\left(m^{2}\right)=\;\sqrt{\frac{Ht\;\left(cm\right)\;\times \;BW\;\left(Kg\right)}{3600}}$$

(2) $$CL_{Cr}\;\left(mL/min/1.73\;m^{2}\right)=\;\frac{k\;\times \;Ht\;\left(cm\right)}{SCr\;\left(mg/dL\right)}$$

### Drug administration and blood sampling

Intravenous doses of piperacillin-tazobactam were based on the piperacillin component (100 mg/kg), with tazobactam administered in a fixed 1:8 ratio (12.5 mg/kg). Dosing frequency was determined according to PMA and PNA as recommended by Neofax: neonates with PMA ≤29 weeks and PNA ≤28 days received 100 mg/kg every 12 h; PMA 30–36 weeks and PNA ≤14 days, 100 mg/kg every 12 h; PMA 30–36 weeks and PNA >14 days, 100 mg/kg every 8 h; PMA 37–44 weeks and PNA ≤7 days, 100 mg/kg every 12 h; and PMA 37–44 weeks and PNA >7 days, 100 mg/kg every 8 h (17). The infusion duration (0.5 or 1 h) was individualized by the nursing staff based on clinical considerations, such as concurrent medications and the characteristics of the venous access. The piperacillin-tazobactam (Piptabac, Pisa) formulation used in this study was a lyophilized powder containing 4 g of piperacillin and 0.5 g of tazobactam.

A sparse sampling strategy was employed to optimize pharmacokinetic data collection while minimizing invasiveness in the neonatal population. The sampling methodology, previously described by our research group, involved collecting two to three opportunistic blood samples of 250 µL each during routine clinical care (63). All samples were immediately protected from light, transferred on ice to the laboratory, and centrifuged at 11,000 rpm for 15 min. The resulting plasma was stored at –80°C for up to 30 days prior to quantification using ultra-high-performance liquid chromatography with tandem mass spectrometry (UPLC-MS/MS).

### Analytical method

Total plasma piperacillin and tazobactam concentrations were quantified using a UPLC-MS/MS method, with dicloxacillin as the internal standard (64). The internal standard was prepared by dissolving dicloxacillin in acetonitrile (10 µg/mL), and 100 µL of this solution was added to 50 µL of plasma, and the obtained mixture was centrifuged at 14,000 rpm for 20 min at 4°C. Then, 100 µL of the supernatant was recentrifuged for 10 min, and 50 µL was diluted with water (1:1, vol/vol). Finally, 5 µL of the final dilution was injected into the UPLC system.

Chromatographic separation was performed on a Waters Acquity HSS T3 column (100 Å, 1.8 µm, 2.1 × 100 mm) at 35°C. The mobile phase consisted of 0.1% formic acid in water (A) and acetonitrile (B), delivered at a flow rate of 0.2 mL/min under a gradient elution program. The injection volume was 5 µL. The analytical method was fully validated in accordance with applicable US Food and Drug Administration guidance for bioanalysis, covering a concentration range of 0.6 to 100 mg/L for piperacillin and 0.6 to 72 mg/L for tazobactam (65).

Population pharmacokinetic modeling was conducted exclusively for piperacillin, as it guides dose optimization. Tazobactam exposure was evaluated descriptively by summarizing plasma concentrations with measures of central tendency and dispersion. Additionally, concentration–time profiles were plotted to examine the distribution of tazobactam plasma concentrations across patients, considering that its dose is fixed and not subject to individual adjustment. Moreover, the piperacillin-tazobactam plasma concentration ratio was calculated for each subject to describe the observed relationship between the two components in this population.

### Pharmacokinetic analysis

#### Base model

Population pharmacokinetic modeling was performed using nonlinear mixed-effects analysis in NONMEM (version 7.4, Icon Development Solutions, Ellicott City, MD, USA) with the first-order conditional estimation method with interaction. Subroutines ADVAN1 TRANS2 and ADVAN3 TRANS4 were tested to evaluate one- and two-compartment models, respectively. The fixed-effect parameters included CL and V for the one-compartment model, and CL, central volume of distribution (V1), peripheral volume (V2), and intercompartmental clearance (Q) for the two-compartment model.

IIV was assessed for CL and V. Residual unexplained variability was evaluated by testing additive, proportional, and combined error models. Potential correlations among random effects were assessed using a parsimonious covariance block structure, retaining only those supported by the data. 

#### Covariate model building

After developing the base model for piperacillin, potential covariates were explored to evaluate their influence on pharmacokinetic parameters. The set of covariates was defined based on previously published pharmacokinetic studies in neonates, known clinical factors influencing drug disposition, and the pharmacological characteristics of piperacillin (27, 28, 37, 39). A graphical exploration of continuous covariates (PNA, GA, PMA, BW, Ht, BSA, SCr, CL_cr_, total bilirubin, total protein, albumin, AST, and ALT) and categorical covariates (sex, mechanical ventilation, sepsis, and co-administration with vancomycin, levetiracetam, caffeine, or paracetamol) was performed to visualize the potential relationship with pharmacokinetic parameters. All covariates were tested for inclusion in the model using a stepwise forward inclusion and backward elimination approach.

Firstly, the influence of BW on piperacillin pharmacokinetics was evaluated using allometric scaling, based on the following Equation 3:


(3) $$\theta_{i}=\theta_{1}\times \;{\left(\frac{BW}{BW\;median}\right)}^{k}$$

In this equation, $\theta_{i}$ represents the individual pharmacokinetic parameter (CL or V), $\theta_{1}$ is the typical population value of the parameter, and k refers to the allometric exponent, which was fixed to 0.75 for CL and 1 for V (66, 67).

The influence of the physiological maturation process, an inherent and dynamic characteristic of neonates, on CL was explored by incorporating PMA using a sigmoidal maturation function, also known as the Hill equation, as shown in Equation 4:


(4) $$F_{PMA}=\;\frac{PMA^{Hill}}{PMA^{Hill}+\;TM_{50}^{Hill}}$$

$F_{PMA}$ represents the maturation factor as a function of PMA. $TM_{50}$ corresponds to the PMA at which piperacillin CL reaches 50% of its mature value. The Hill coefficient describes the slope of the maturation curve, characterizing how rapidly CL approaches the mature level as PMA increases.

Due to the limited number of patients and the sparse sampling design, it was not feasible to estimate the TM_50_ and Hill coefficient parameters reliably in our study population. Therefore, alternative strategies were considered to define these values. One approach was to adopt the estimates reported by De Cock et al. (68) from a pharmacokinetic study of piperacillin conducted in pediatric patients (PNA: 2 months to 15 years), in which TM_50_ was 61.2 weeks and the Hill coefficient was 1.62 (68). An additional strategy involved using the values proposed by Rhodin et al. (35) to characterize the maturation of glomerular filtration (TM_50_ = 47.7 weeks; Hill coefficient = 3.4), given that renal CL represents the primary elimination pathway of piperacillin (35).

To evaluate the inclusion of continuous covariates, linear (Equation 5), allometric (Equation 6), and exponential (Equation 7) functions were tested, where $\theta_{i}$ represents the individual pharmacokinetic parameter value, and $\theta_{1}$ the typical population value. The term $\theta_{2}$ quantifies the magnitude and direction of the covariate effect on the parameter of interest. In all cases, covariates (COV) were centered around their median values (${COV}_{median})$) to improve interpretability and numerical stability.


(5) $$\theta_{i}=\theta_{1}\times \;\left(1+\;\theta_{2}\times \frac{COV-COV\;median}{COV\;median}\;\right)$$

(6) $$\theta_{i}=\theta_{1}\times \;{\left(\frac{COV}{COV\;median}\right)}^{\theta_{2}}$$

(7) $$\theta_{i}=\theta_{1}\times \;exp\left(\theta_{2}\times \frac{COV-COV\;median}{COV\;median}\;\right)$$

To explore the inclusion of the categorical covariates, the implementation of the covariates in the model was tested by Equation 8:


 (8) $$\theta_{i}=\theta_{1}\times \;\left(1+\;\theta_{2}\times COV\;\right)$$

To prevent multicollinearity, correlated covariates describing the same maturational process, such as PMA, PNA, and GA, were not evaluated simultaneously. Covariate inclusion was based on a statistically significant reduction in objective function value (ΔOFV ≥ 3.84 per degree of freedom, *P* < 0.05), assuming a chi-squared distribution for nested models, and supported by goodness-of-fit statistics, along with diagnostic goodness-of-fit plots. Backward elimination was performed on the full model once no further improvement was observed; covariates were removed in the reverse order of inclusion and retained in the final model if their removal caused a ΔOFV > 6.63 per degree of freedom (*P* < 0.01). Covariates that significantly reduced IIV and improved the fixed-effect estimates were retained (69, 70).

### Model evaluation

Internal model evaluation was performed to assess parameter robustness and predictive performance. Initially, goodness-of-fit plots were used for diagnostic purposes. Model stability was evaluated using bootstrap analysis, and predictive accuracy was assessed using a pcVPC and NPDEs based on 1,000 simulated individuals. All procedures were executed in NONMEM, with Pirana (version 2.9.8) as the interface and PsN (version 5.2.6) for post-processing. NPDE simulations were exported to R (version 4.2.2) for analysis using the NPDE package (version 3.4) (71).

External validation was conducted using an independent data set comprising 17 plasma samples from eight patients in our clinical cohort, which were *a priori* set aside for validation and not included in model development. Predicted piperacillin plasma concentrations were compared with observed values, and model performance was evaluated in terms of predictive accuracy and precision using the MPE, MAPE, and RMSE, both in absolute values and as percentages. Additionally, a pcVPC was also performed using the external data set to provide a graphical evaluation of the final model’s predictive performance.

### Monte Carlo simulations for evaluation and optimization of dosing regimens

The final population pharmacokinetic model for piperacillin was used to perform Monte Carlo simulations to evaluate current dosing recommendations in neonates and to explore optimized strategies across clinically relevant subgroups. Two commonly used regimens, as recommended in Neofax (17) and the Harriet Lane Handbook (18) (Table 6), were selected and simulated in virtual neonatal populations generated using NONMEM.

**TABLE 6:** Neonatal piperacillin-tazobactam dosing regimens evaluated in the present study^*a*^

Regimen	PMA (weeks)	PNA (days)	BW (kg)	SCr (mg/dL)	Dose (mg/kg)	Interval (h)	Infusion duration (h)
The Harriet Lane Handbook (18)	(All)	≤7	≤2		100	8	0.5
	≤30	>7	≤2		100	8	0.5
	>30	>7	≤2		80	6	0.5
	≤35	≤28	>2		80	6	0.5
	>35	≤28	>2		80	4	0.5
Neofax (17)	≤29	≤28			100	12	0.5
	30−36	≤14			100	12	0.5
	30−36	>14			100	8	0.5
	37−44	≤7			100	12	0.5
	37−44	>7			100	8	0.5
Dosing optimization simulations	≤29	≤28		<0.4	30−100	8, 12	0.5, 1, 2, 3, 4
	≤29	≤28		0.4−0.8	30−100	8, 12	0.5, 1, 2, 3, 4
	≤29	≤28		>0.8−1.2	30−100	8, 12	0.5, 1, 2, 3, 4
	30−36	≤28		<0.4	50−120	8, 12	0.5, 1, 2, 3, 4
	30−36	≤28		0.4−0.8	50−120	8, 12	0.5, 1, 2, 3, 4
	30−36	≤28		>0.8−1.2	50−120	8, 12	0.5, 1, 2, 3, 4
	37−44	≤28		<0.4	100−130	8, 12	0.5, 1, 2, 3, 4
	37−44	≤28		0.4−0.8	100−130	8, 12	0.5, 1, 2, 3, 4
	37−44	≤28		>0.8−1.2	100−130	8, 12	0.5, 1, 2, 3, 4

^*a*^ Only the dosing criteria applicable to neonates (0−28 days of postnatal age) were extracted from each original regimen. PMA: Postmenstrual age; PNA: postnatal age; BW: body weight; SCr: serum creatinine; Interval: dosing interval.

Neonates were stratified into three groups according to PMA: extremely preterm (≤29 weeks), moderate to late preterm (30−36 weeks), and term (≥37 weeks). For each group, 100 virtual patients were generated using empirical distributions and observed correlations between PMA, birth weight, and SCr from the clinical data set. For the extremely preterm group, which included only one patient in the observed data, covariate values were defined using study inclusion criteria, literature sources, and reference weights from the Fenton growth charts (41, 72). For each virtual individual, 100 unbound piperacillin concentration–time profiles were simulated, assuming 30% plasma protein binding (48).

The PK-PD target (fT>MIC) was evaluated for the CLSI-recommended susceptibility breakpoints for Gram-negative pathogens relevant to neonatal infections (72). Two PK-PD thresholds were considered: a conservative ≥50% fT>MIC, and a stricter ≥75% fT>MIC, based on previous reports (25, 26, 28). The probability of target attainment was calculated for each threshold. Additionally, a trough piperacillin concentration of 50 mg/L was used as an exploratory safety threshold, as it has been associated with an increased risk of acute kidney injury in the pediatric population (54).

Optimized dosing strategies were explored using model-based simulations in each PMA group. Representative virtual patients were stratified by SCr into three categories: <0.4 mg/dL, 0.4–0.8 mg/dL, and >0.8–1.2 mg/dL. These categories were selected based on a sensitivity analysis of the impact of SCr on piperacillin plasma concentrations. This approach aims to improve clinical applicability and to prevent excessive dose adjustments for the dynamic changes in renal function that are inherent in neonates (73, 74).

Simulated intravenous piperacillin doses ranged from 30 to 130 mg/kg administered every 8 or 12 h. Extended infusion durations from 1 to 4 h were also evaluated (75). Optimization focused on achieving ≥75% fT>MIC, while trough concentrations were assessed to identify potential overexposure risks related to nephrotoxicity.

### Conclusion

A one-compartment population pharmacokinetic model for piperacillin was developed exclusively in neonates (PNA ≤28 days), identifying BW, PMA, and SCr as significant covariates influencing drug CL and highlighting the critical role of renal function in piperacillin disposition. The model demonstrated robust predictive performance, supported by both internal and external validation using neonatal data. This study provides guidance on initial dosing in neonates with similar clinical features, aiming to balance effectiveness and safety. However, clinical studies evaluating efficacy and toxicity are necessary prior to implementation.

## Data Availability

Data from this study cannot be shared publicly, due to the data privacy statement included in the consent documents signed by the parents or legal guardians of the patients involved in this research.

## SUPPLEMENTAL MATERIAL

Supplemental Figures and Tables aac.00998-25-s0001.pdf Table S1 and Figures S1–S3.

ASM does not own the copyrights to Supplemental Material that may be linked to, or accessed through, an article. The authors have granted ASM a non-exclusive, world-wide license to publish the Supplemental Material files. Please contact the corresponding author directly for reuse.
