# Supplementary material for: Population pharmacokinetics and dose optimization of piperacillin-tazobactam in premature and term neonates with severe infections
Source: Antimicrob Agents Chemother. 2025 Nov 25;70(1):e00998-25. doi: 10.1128/aac.00998-25 (PMC12777573; doi:10.1128/aac.00998-25)
Supplement: Supplemental Figures and Tables — Table S1 and Figures S1–S3. [file aac.00998-25-s0001.pdf]

Supplementary Material

Population pharmacokinetics and dose optimization of piperacillin-tazobactam in premature and term neonates with severe infections.

Antimicrobial Agents and Chemotherapy.

Running Head: Population Pharmacokinetics of piperacillin-tazobactam in neonates.

Frida S. Boer-Pérez[a], Victoria Lima-Rogel[b], Silvia Romano-Moreno[a], Ana R. Mejía-Elizondo[b], Susanna E. Medellín-Garibay[a], Paula Schaiquevich[c,d], Daniel E. Noyola-Cherpitel[e], Ana S. Rodríguez-Báez[f], Cristian J. Rodríguez-Pinal[a], Rosa del C. Milán-Segovia[a]#.

[a]Facultad de Ciencias Químicas, Universidad Autónoma de San Luis Potosí, San Luis Potosí, Mexico.

[b]Neonatal Intensive Care Unit, Hospital Central "Dr. Ignacio Morones Prieto", San Luis Potosí, Mexico.

[c]Unit of Innovative Treatments, Hospital de Pediatria JP Garrahan, Buenos Aires CP1245, Argentina

[d]National Council of Scientific and Technical Research (CONICET), Buenos Aires C1425 FQB, Argentina

[e]Microbiology Department, Facultad de Medicina, Universidad Autónoma de San Luis Potosí, San Luis Potosí, Mexico

[f]Department of Clinical Pharmacy, Institute of Pharmacy, University of Bonn, Bonn, Germany.

# Address correspondence to Rosa del C. Milán-Segovia, PharmD

milanros@uaslp.mx

phone: +52 444-826-2440 ext. 6513

Facultad de Ciencias Químicas, Universidad Autónoma de San Luis Potosí, Manuel Nava Martínez Ave. #6, University Zone, San Luis Potosí, 78210, Mexico.

Contents

Tabla S1: Significant steps in piperacillin population PK model-building process. 2

Figure S1. Piperacillin (A) and tazobactam (B) total plasma concentration versus time after dose plots. ..2

Figure S2. Piperacillin-tazobactam ratios over time in neonatal patients. 3

Figure S3. Prediction-corrected visual predictive check (pcVPC) for the external neonatal cohort (n=8) based on 1,000 simulations...4

Tabla S1: Significant steps in piperacillin population PK model-building process.

Pop-PK Model	CL (L/h)	ω_{CL} (%CV)	V (L)	ω_V (%CV)	OFV	ΔOFV
Base model	0.202	85.0	0.956	53.0	530.695	
$CL = \theta_{CL} * \left(\frac{BW}{1.76}\right)^{0.75}$	0.198	67.5	0.954	57.1	515.116	15.579
$CL = \theta_{CL} * \left(\frac{BW}{1.76}\right)^{0.75} * \frac{PMA^{3.4}}{PMA^{3.4} + 47.7^{3.4}}$	0.711	44.8	0.953	57.6	497.876	17.240
$CL = \theta_{CL} * \left(\frac{BW}{1.76}\right)^{0.75} * \frac{PMA^{3.4}}{PMA^{3.4} + 47.7^{3.4}} * \left(\frac{SCr}{0.4}\right)^{-0.635}$	0.740	38.6	0.957	56.6	491.099	6.777
$V = \theta_V * \left(\frac{BW}{1.76}\right)^{1}$	0.748	38.3	0.866	37.7	474.888	16.211

CL: clearance; BW: body weight; PMA: postmenstrual age; SCr: serum creatinine; V: volume of distribution; ω_{CL}: interindividual variability of clearance; ω_V: interindividual variability of volume of distribution; OFV: objective function value.

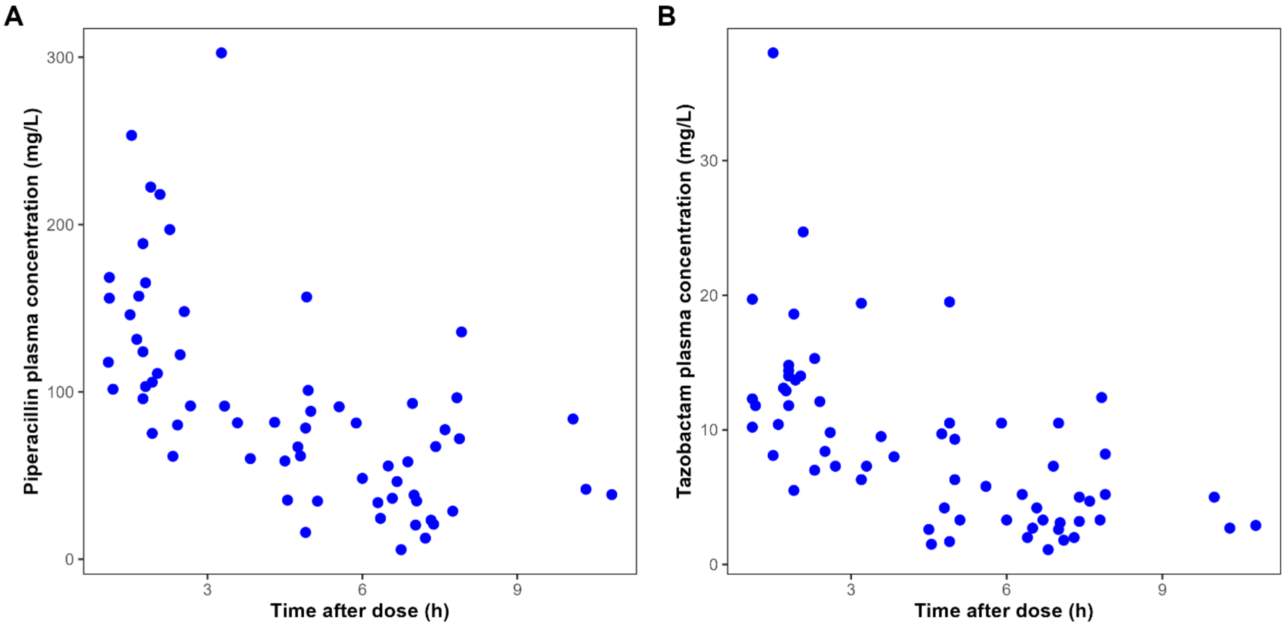

Figure S1. Piperacillin (A) and tazobactam (B) total plasma concentration versus time after dose plots. Blue circles represent observed plasma concentrations in neonatal patients.

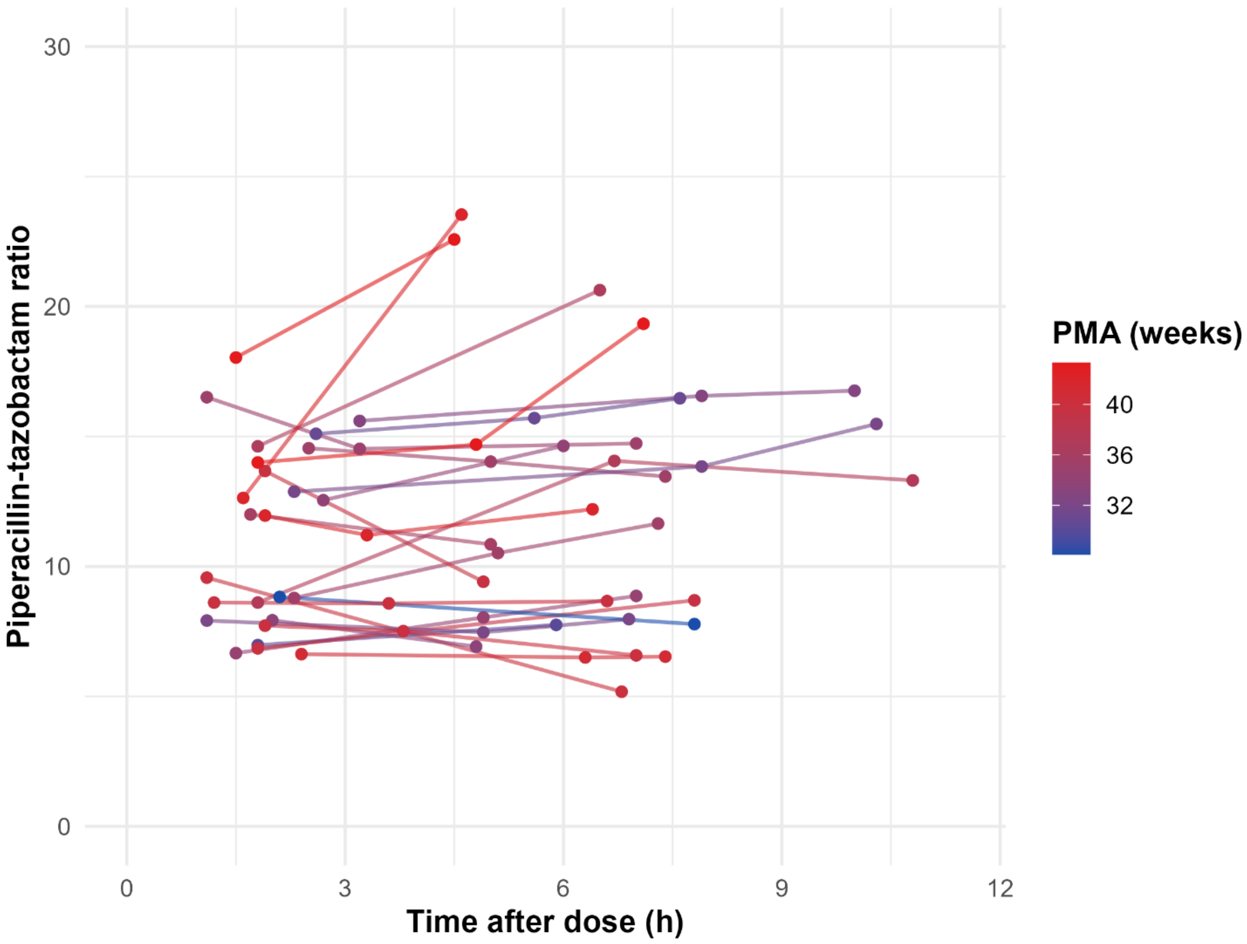

Figure S2. Piperacillin-tazobactam ratios over time in neonatal patients. Each line represents an individual patient, and points indicate measured ratios at each time point. Colors correspond to postmenstrual age (PMA) in weeks, with blue indicating lower PMA and red indicating higher PMA.

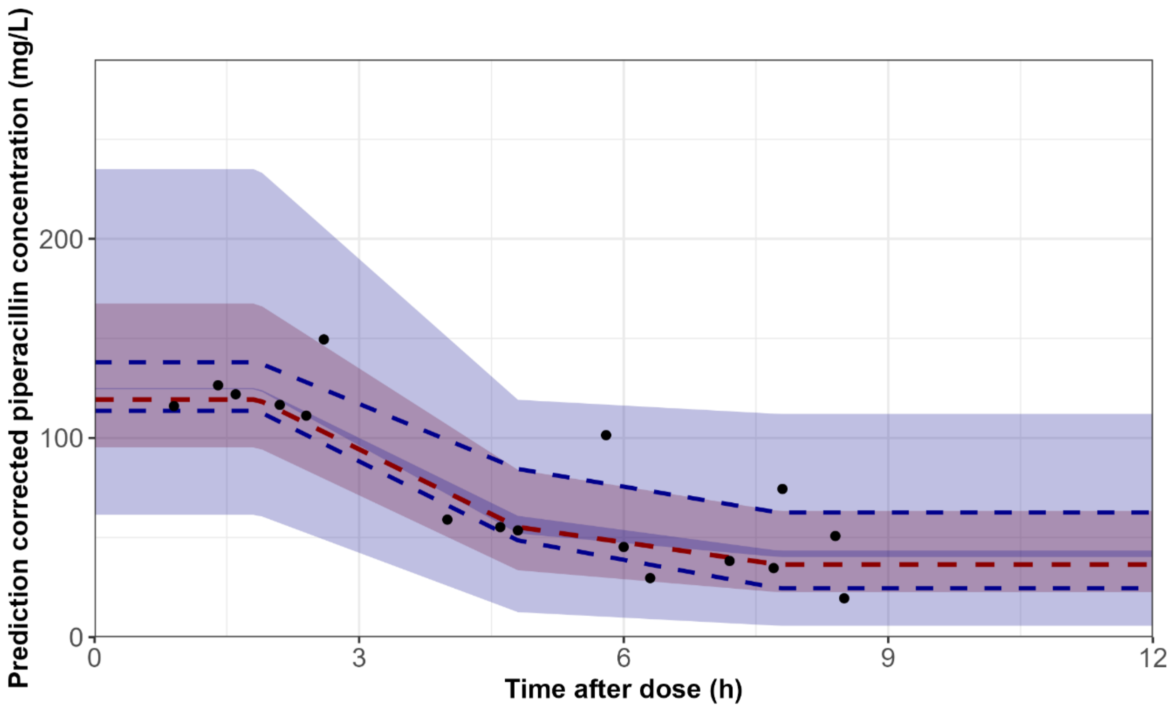

Figure S3. Prediction-corrected visual predictive check (pcVPC) for the external neonatal cohort (n=8) based on 1,000 simulations. Black circles represent prediction-corrected observed piperacillin plasma concentrations. Dashed lines indicate the observed median (red) and 10th and 90th percentiles (blue). Shaded areas show the 95% confidence intervals of the simulated median (red) and percentiles (blue).
